# Reversal of Type 2 Diabetes in a Patient With Hashimoto’s Thyroiditis Through Combined Pharmacologic and Lifestyle Intervention

**DOI:** 10.7759/cureus.85820

**Published:** 2025-06-12

**Authors:** Sherif I Ramoun, Sherif A Zaki, Amr M Issa, Ahmed K Attalla

**Affiliations:** 1 Internal Medicine/Pediatrics, Alexandria University Main Hospitals, Alexandria, EGY; 2 Dermatology, Alexandria University, Alexandria, EGY; 3 Internal Medicine, Faculty of Medicine, Alexandria University, Alexandria, EGY; 4 Intensive Care Unit, Alexandria University Main Hospitals, Alexandria, EGY

**Keywords:** autoimmune hypothyroidism, calorie restriction, hashimoto’s thyroiditis, lifestyle intervention, metformin withdrawal, resistance training, type 2 diabetes remission

## Abstract

Type 2 diabetes mellitus (T2DM) is typically considered a progressive condition, but recent evidence suggests potential reversibility through structured lifestyle intervention. We report the case of a 32-year-old Egyptian female with Hashimoto’s thyroiditis and newly diagnosed T2DM, confirmed by elevated fasting blood glucose and HbA1c levels. The patient was managed with once-daily metformin, continued levothyroxine, a 500-calorie daily energy deficit, and progressive resistance training three times per week. After 12 months, fasting glucose and HbA1c values decreased below the diagnostic thresholds for diabetes, allowing metformin to be discontinued. Follow-up at one, three, and six months post-intervention confirmed sustained biochemical remission. This case highlights the potential for reversing diabetes in select patients through a combined pharmacological and lifestyle approach.

## Introduction

Type 2 diabetes mellitus (T2DM) is one of the most common chronic conditions globally, often viewed as a progressive disease requiring long-term medical treatment. However, recent evidence has shown that with early and targeted intervention, remission is possible in certain patients [1].

Lifestyle changes, particularly those involving weight loss and physical activity, play a key role in achieving this outcome, while in clinical practice, managing diabetes becomes even more challenging when it coexists with other endocrine disorders such as Hashimoto’s thyroiditis, which can affect insulin sensitivity and complicate metabolic control [1].

In this report, we describe the case of a 32-year-old woman with both T2DM and Hashimoto’s thyroiditis who was able to achieve and maintain remission of her T2DM through a combination of low-dose metformin (250 mg daily) treatment, dietary modification, and consistent resistance training. This case adds to the growing body of evidence supporting the potential for diabetes reversal in carefully selected individuals.

## Case presentation

A 32-year-old Egyptian female with a known history of Hashimoto’s thyroiditis, managed with levothyroxine, presented for evaluation of elevated fasting blood glucose levels. She reported mild fatigue but no classic hyperglycemia symptoms such as polyuria or polydipsia. There was no family history of diabetes, and her body mass index (BMI) was consistent with an overweight status, though body composition was not formally assessed. Laboratory investigations revealed a fasting blood glucose of 7.1 mmol/L and an HbA1c of 49 mmol/mol. Both readings were confirmed with repeat testing over the following weeks, meeting the diagnostic criteria for type 2 diabetes mellitus (fasting blood glucose of >6.9 mmol/L and HbA1c of >48 mmol/mol).

After discussing treatment options, we initiated a once-daily dose of metformin and maintained her existing levothyroxine regimen. In parallel, the patient agreed to adopt a 500-calorie daily energy deficit and begin a progressive resistance training program three times per week. This multidisciplinary approach aimed to address both insulin sensitivity and weight-related metabolic factors, as supported by findings from recent diabetes remission trials.

After 12 months of adherence, repeat labs showed improvement: her fasting glucose had decreased to 6.5 mmol/L and HbA1c to 44 mmol/mol - no longer within the diabetic range. Given her progress and clinical stability, metformin was discontinued. Follow-up testing at one, three, and six months after stopping the medication confirmed sustained remission. Her fasting glucose consistently remained under 6.9 mmol/L, and HbA1c ranged between 42 and 45 mmol/mol, supporting durable remission in the absence of pharmacotherapy (Table 1).

**Table 1:** Laboratory results before and after intervention and post-metformin discontinuation. Reference ranges are provided for clinical interpretation. TSH, thyroid-stimulating hormone; HbA1c, glycated hemoglobin; LDL, low-density lipoprotein

Test	Baseline	12 months	6 months post-metformin	Reference range
Fasting blood glucose	7.1 mmol/L	6.5 mmol/L	6.3 mmol/L	3.9-5.5 mmol/L
HbA1c	49 mmol/mol	44 mmol/mol	42-45 mmol/mol	<42 mmol/mol
TSH	6.5 mIU/L	3.2 mIU/L (stable)	3.1 mIU/L	0.4-4.0 mIU/L
LDL cholesterol	3.5 mmol/L	2.6 mmol/L	2.4 mmol/L	<2.6 mmol/L
Total cholesterol	5.5 mmol/L	4.7 mmol/L	4.6 mmol/L	<5.2 mmol/L
Triglycerides	2.0 mmol/L	1.5 mmol/L	1.3 mmol/L	<1.7 mmol/L

## Discussion

The remission of T2DM has gained increasing interest in recent years, particularly through lifestyle-based strategies targeting weight reduction, improved insulin sensitivity, and metabolic homeostasis [1]. In this case, a combination of modest pharmacologic support with metformin, structured caloric restriction, and regular resistance training resulted in sustained glycemic control, eventually allowing for discontinuation of medication. Her outcome aligns with current evidence indicating that intensive lifestyle intervention can reverse T2DM in select individuals [1-3].

This case is particularly noteworthy because of the coexistence of Hashimoto’s thyroiditis, an autoimmune thyroid disorder known to contribute to reduced insulin sensitivity and increased cardiometabolic risk. Although hypothyroidism does not directly cause T2DM, it can impair glucose utilization and exacerbate metabolic dysfunction. Prior studies have shown that autoimmune thyroid disease may be associated with poorer glycemic control and a diminished response to lifestyle interventions [4-5]. Nonetheless, this patient’s achievement of remission despite such a comorbidity demonstrates that early, aggressive intervention can overcome endocrine barriers when implemented consistently [4-5].

The role of resistance training deserves emphasis. Beyond promoting weight loss, resistance exercise improves insulin sensitivity through increased Glucose Transporter Type 4 (GLUT-4) translocation in skeletal muscle and enhances metabolic flexibility [6]. When paired with a hypocaloric diet, it can facilitate physiologic adaptations strong enough to support diabetes remission even in the absence of pharmacotherapy [6,7].

Although metformin was used during the initial phase, the patient’s sustained normoglycemia after its discontinuation suggests that lifestyle modification was the primary driver of remission. This reinforces the position of several professional societies, including the American College of Lifestyle Medicine, which advocates for structured diet and exercise regimens as first-line treatments for early-stage T2DM [6]. Long-term outcomes, however, depend heavily on patient engagement and behavior change factors that are challenging to generalize across broader populations [7].

This case also highlights the importance of individualized care. Rather than relying solely on clinical algorithms or escalation to polypharmacy, the care team focused on empowering the patient with achievable, evidence-based goals. Her success was not the result of a novel medication, but rather a well-executed plan rooted in education, support, and persistence. Future research should continue to explore how such strategies can be scaled and tailored to different patient populations, particularly those with autoimmune comorbidities or limited access to specialty care [8,9,10].

## Conclusions

This case demonstrates that sustained remission of T2DM is achievable through a structured and consistent approach that includes pharmacologic support, nutritional discipline, and physical training.

As supported by emerging research, early intervention using lifestyle-based approaches can offer a powerful alternative to the conventional view of diabetes as an irreversible condition.
